# Splenic Infarction and Recurrent Pulmonary Embolism in Atrial Fibrillation Associated With Subtherapeutic Anticoagulation and Medication Nonadherence: A Case Report

**DOI:** 10.7759/cureus.103276

**Published:** 2026-02-09

**Authors:** Tony Fares, Rauann Hachem, Abshiro Mayow, Muhammad A Shahzad

**Affiliations:** 1 Department of Medicine, St. George's University, St. George, GRD; 2 Department of Medicine, Saint James School of Medicine - Anguilla Campus, The Quarter, AIA; 3 Department of Internal Medicine, AMITA Health Adventist Medical Center, GlenOaks, Glendale Heights, USA

**Keywords:** abdominal pain, anticoagulants, atrial fibrillation, dyspnea, left atrial appendage, pulmonary embolism, splenic infarction, thrombosis

## Abstract

Atrial fibrillation (AF), the most common sustained arrhythmia worldwide, remains a major cause of cardioembolic stroke and systemic thromboembolism due to impaired atrial contraction, blood stasis, and endothelial injury within the left atrial appendage. Although oral anticoagulation with vitamin K antagonists or direct oral anticoagulants substantially reduces thromboembolic risk, maintaining stable and uninterrupted therapeutic exposure remains a persistent clinical challenge. Splenic infarction (SI) represents a rare extracerebral manifestation of systemic embolization and may present with nonspecific abdominal pain, often delaying diagnosis. We report a 60-year-old man with chronic AF maintained on warfarin who presented with acute abdominal pain and was found to have multiple splenic infarcts, pulmonary emboli, and a left atrial appendage thrombus in the setting of recurrent subtherapeutic anticoagulation. During hospitalization, he received parenteral anticoagulation with appropriate transition to apixaban at discharge; however, he did not obtain or initiate the prescribed medication and returned two days later with recurrent pulmonary embolism and persistent symptoms. His readmission was further complicated by pneumonia, likely acquired through household exposure, potentially intensifying a prothrombotic state during a period of anticoagulation instability. By illustrating the consequences of fluctuating anticoagulation control and documented nonadherence, this case reinforces the importance of vigilant therapeutic monitoring, careful transitions between regimens, early imaging in anticoagulated patients with unexplained abdominal pain, and coordinated management strategies aimed at preventing recurrent and potentially life-threatening embolic complications.

## Introduction

The spleen is a highly vascular organ central to immune surveillance, hematologic regulation, and blood filtration [1]. Splenic infarction (SI) is an uncommon but clinically significant condition resulting from arterial occlusion and subsequent ischemic necrosis. Among its etiologies, cardioembolic phenomena, particularly those associated with atrial fibrillation (AF), represent a leading cause [2,3].

Although cerebral embolism remains the most recognized thromboembolic complication of AF, systemic embolization to the spleen is comparatively rare and likely underreported. In a 10-year cohort study, the hospital incidence of SI was estimated at only 0.016%, with cardiogenic emboli accounting for the majority of cases [4]. Clinical presentation is often nonspecific, including left upper quadrant pain, fever, or referred shoulder discomfort, which may delay diagnosis or mimic other intra-abdominal conditions [5,6]. Contrast-enhanced computed tomography remains the diagnostic modality of choice due to its high sensitivity [1,5].

AF affects more than 30 million individuals worldwide and substantially increases the risk of systemic thromboembolism through impaired atrial contraction, blood stasis, and endothelial dysfunction within the left atrial appendage (LAA) [7-10]. These pathophysiologic mechanisms most commonly manifest as ischemic stroke; however, SI remains a rare extracerebral expression of embolic disease despite the global prevalence of AF [4]. This discrepancy suggests that SI may be underrecognized, particularly in patients presenting with vague or nonspecific abdominal symptoms in whom early imaging is not pursued. Oral anticoagulation with vitamin K antagonists or direct oral anticoagulants significantly reduces thromboembolic risk in AF [11-13]. Nevertheless, maintaining stable and uninterrupted therapeutic anticoagulation remains a persistent challenge in clinical practice. Variability in anticoagulant response, fluctuating international normalized ratio (INR) control, and lapses in adherence or monitoring may compromise effective protection against systemic embolization [11,12].

We present the case of a 60-year-old man with chronic AF who developed SI and pulmonary embolism in the setting of recurrent subtherapeutic anticoagulation while maintained on warfarin therapy. His INR at presentation was below the recommended therapeutic range of 2.0-3.0 [13], and prior outpatient laboratory data demonstrated persistent instability. Following inpatient anticoagulation and transition to apixaban at discharge, documented nonadherence preceded readmission with recurrent pulmonary embolism. This case illustrates the clinical consequences of anticoagulation instability and nonadherence, emphasizes the importance of early imaging in AF patients with unexplained abdominal pain, and supports individualized strategies aimed at maintaining therapeutic continuity in high-risk populations. We present this case in accordance with the Case Report (CARE) guidelines.

## Case presentation

A 60-year-old man with hypertension, long-term tobacco use, and persistent AF, chronically managed with warfarin 5 mg daily, presented to the emergency department with sudden, severe abdominal pain that began approximately one hour before arrival. The pain was initially epigastric and later became diffuse. He reported mild nausea but denied vomiting, fever, chills, diarrhea, or urinary symptoms. He had been discharged two days earlier from Central DuPage Hospital after a five-day admission for pneumonia and influenza B and was sent home on supplemental oxygen without antibiotics. On admission, laboratory evaluation demonstrated a subtherapeutic international normalized ratio (INR) of 1.25 (therapeutic range 2.0-3.0 for AF). During inpatient monitoring, a repeat INR was documented at 1.98, which remained below the therapeutic target. Prior outpatient laboratory values had also shown recurrent subtherapeutic anticoagulation. No documented contraindications to direct oral anticoagulants were identified in the medical record. He denied prior venous thromboembolism, malignancy, or known bleeding disorders, and there was no family history of thrombosis. He was a former smoker with more than 20 pack-years.

On examination, he was alert and fully oriented with a normal mental status. Initial vital signs were blood pressure 139/82 mmHg, heart rate 104 beats per minute, respiratory rate 16 breaths per minute, temperature 36.7 °C (98.1 °F), and oxygen saturation 97% on room air. He remained hemodynamically stable during the emergency department course. The abdomen was diffusely tender without guarding, rebound, or distension. Cardiac examination noted an irregular rhythm. The lungs were clear to auscultation bilaterally. Neurologic examination was nonfocal.

**Figure 1:**
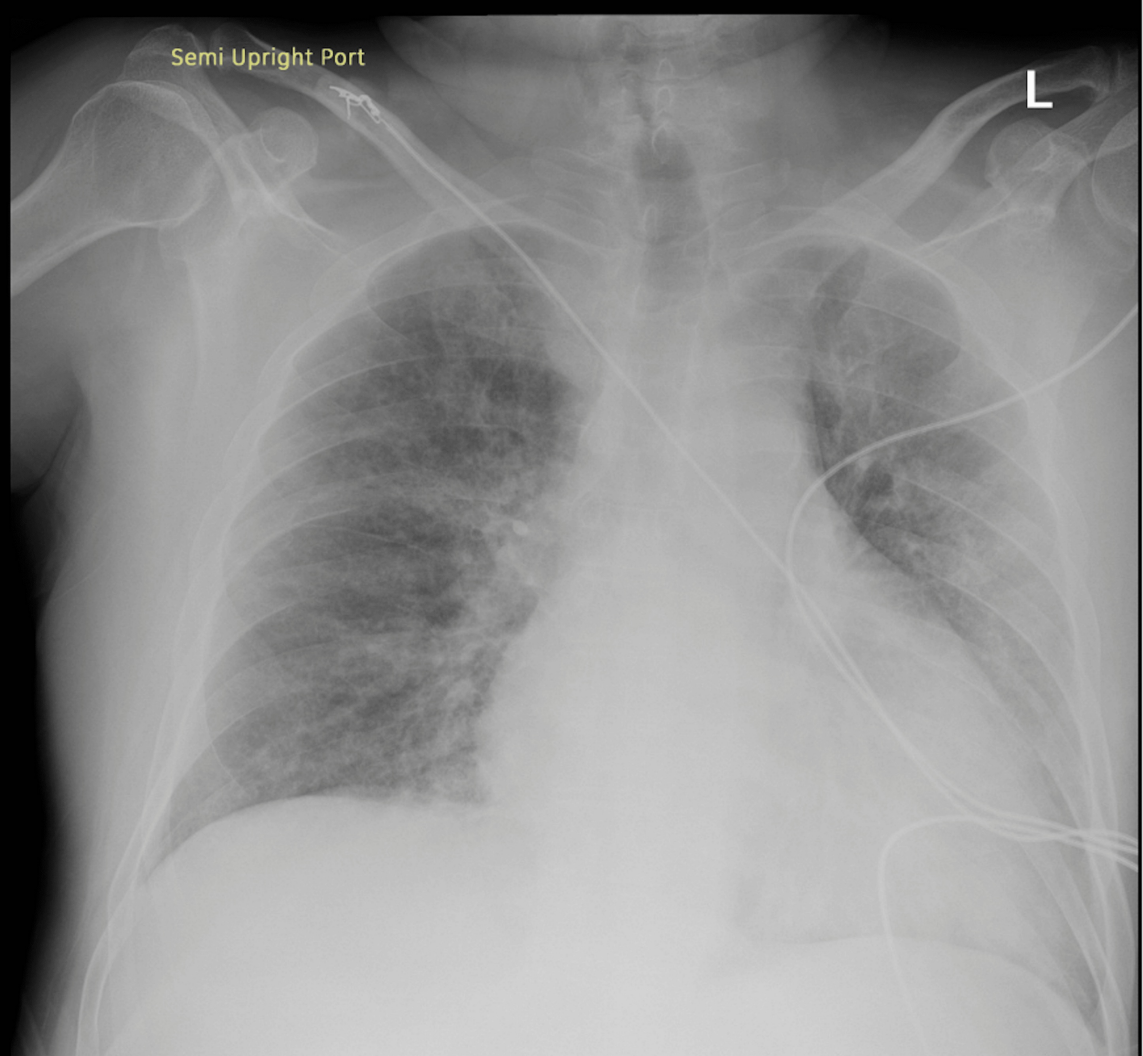
Chest radiograph (AP view) demonstrating diffuse interstitial opacities bilaterally and an enlarged cardiac silhouette, findings suggestive of congestive heart failure versus residual infectious changes.

**Figure 2:**
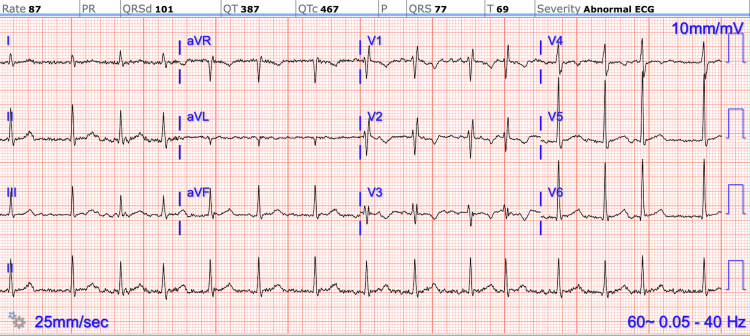
Twelve-lead ECG demonstrating atrial fibrillation with an irregularly irregular rhythm, absence of discrete P waves, and an incomplete right bundle branch block pattern. The QRS duration was prolonged at 101 ms (≥90 ms, <120 ms), with a terminal R′ deflection visible in leads V1–V2.

A chest radiograph revealed diffuse interstitial opacities and cardiomegaly, raising concern for congestion versus residual infectious infiltrates (Figure 1). Electrocardiography showed AF with an incomplete right bundle branch block pattern; the QRS duration was 101 ms with a terminal R′ in leads V1-V2 (Figure 2). Laboratory testing demonstrated mild leukocytosis (white blood cells 12.55 × 10⁹/L), mild anemia (hemoglobin 12.2 g/dL), hyponatremia (sodium 132 mmol/L), hypocalcemia (calcium 8.0 mg/dL), and mildly elevated aminotransferases (aspartate aminotransferase 44 U/L, alanine aminotransferase 63 U/L). Prothrombin time was 22.5 seconds, activated partial thromboplastin time 24 seconds, and international normalized ratio (INR) 1.25. B-type natriuretic peptide (BNP) was mildly elevated at 156.4 pg/mL. Troponin and urinalysis were within normal limits. Blood cultures were obtained at presentation, and serum lactate was 1.3 mmol/L. Laboratory results on the day of admission are shown in Table 1.

**Table 1:** Laboratory results on admission and two days after discharge WBC: white blood cell; Hgb: hemoglobin; Na: sodium; Ca: calcium; AST: aspartate aminotransferase; ALT: alanine aminotransferase; PT: prothrombin time; aPTT: activated partial thromboplastin time; INR: international normalized ratio; BNP: B-type natriuretic peptide; hs-cTnI: high-sensitivity cardiac troponin I; TSH: thyroid-stimulating hormone

Parameter	On admission	Two days after discharge	Reference range
WBC count (×10⁹/L)	12.55	16.25	4.0–11.0
Hgb (g/dL)	12.2	12.4	13.5–17.5
Na (mmol/L)	132	127	135–145
Ca (mg/dL)	8.0	8.4	8.5–10.5
AST (U/L)	44	60	10–40
ALT (U/L)	63	66	7–56
PT (seconds)	22.5	18.3	11–13.5
aPTT (seconds)	24	26	25–35
INR	1.25	1.46	0.8–1.2
BNP (pg/mL)	156.4	128.6	<100
Serum lactate (mmol/L)	1.3	1.4	0.5–2.2
hs-cTnI (ng/L)	3.4	3.4	<14
Urinalysis	Normal	Trace protein; occasional bacteria	Negative for protein/bacteria
TSH (μIU/mL)	2.0	7.849	0.4–4.0
Lipase (U/L)	21	30	0–160

**Figure 3:**
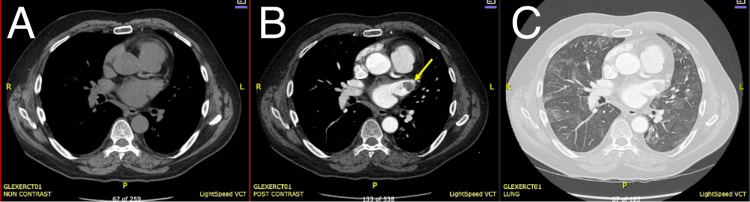
Computed tomographic angiography (CTA) of the chest. Panel A: Non-contrast axial view of the chest. Panel B: Post-contrast axial view demonstrating a lobulated 3-cm filling defect in the LAA (yellow arrow), consistent with thrombus. Panel C: Lung window axial view showing scattered segmental pulmonary emboli.

**Figure 4:**
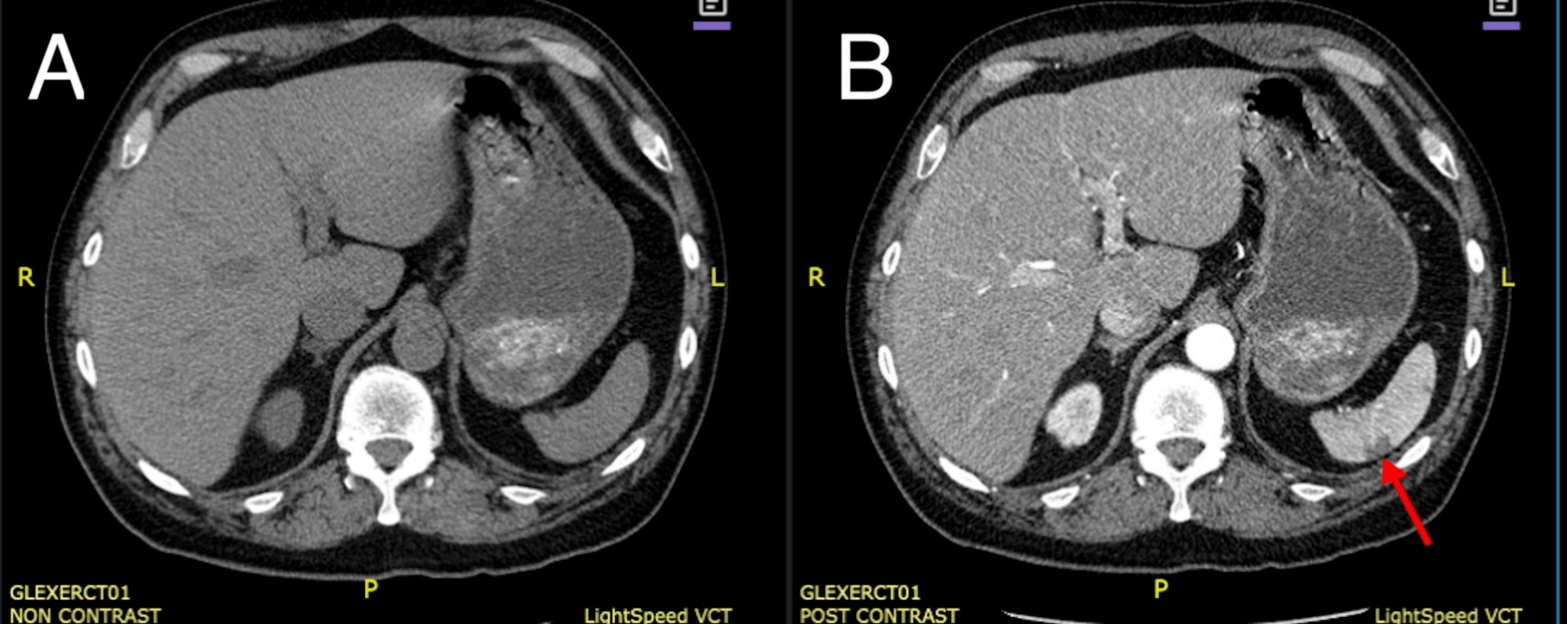
Computed tomography scan. Panel A: without contrast. Panel B: with contrast, demonstrating multiple areas of splenic infarction (red arrow).

Computed tomographic angiography (CTA) of the chest demonstrated scattered segmental pulmonary emboli and a lobulated 3-cm filling defect in the LAA, consistent with thrombus (Figure 3). Bilateral lower-extremity venous ultrasonography showed no evidence of thrombosis in the common femoral, femoral, proximal deep femoral, or popliteal veins bilaterally. D-dimer was 9.18 μg/mL. Contrast-enhanced CT of the abdomen and pelvis showed multiple wedge-shaped, peripheral hypodensities in the spleen, consistent with splenic infarcts (Figure 4).

Anticoagulation was initiated with intravenous unfractionated heparin upon admission due to subtherapeutic INR and evidence of systemic embolism. Warfarin 5 mg daily was resumed with parenteral anticoagulation overlap, and therapeutic dose enoxaparin (80 mg subcutaneously) was administered during bridging. Analgesia included morphine, fentanyl, and hydromorphone, resulting in gradual improvement of abdominal pain. Blood cultures later grew gram-positive bacilli, and empiric piperacillin-tazobactam was initiated for suspected residual pneumonia. Internal medicine, hematology, and cardiology were consulted for multidisciplinary management. The patient remained hemodynamically stable throughout hospitalization. By hospital day four, he reported significant symptom improvement and was transitioned from warfarin to apixaban, initiated at 10 mg twice daily for seven days, followed by 5 mg twice daily for long-term anticoagulation. He was discharged with close outpatient follow-up and rate control therapy, including diltiazem extended release 360 mg once daily and metoprolol succinate 25 mg once daily.

Although admission INR values were below target, prior laboratory data demonstrated recurrent subtherapeutic anticoagulation, suggesting prolonged instability. Thrombus formation may occur during such periods of inadequate anticoagulation and may not be immediately mitigated by subsequent therapeutic INR values.

Two days after discharge, he returned with persistent abdominal pain and new shortness of breath. He reported that he had not yet obtained his prescribed medications. He also reported possible infectious exposure from his spouse, a housekeeper with frequent contact with ill individuals. On arrival, vital signs were heart rate 103 beats per minute, blood pressure 119/70 mmHg, temperature 36.1 °C (97.0 °F), and an irregular cardiac rhythm. Laboratory testing showed worsening leukocytosis (WBC 16.25 × 10⁹/L), mild anemia (Hgb 12.4 g/dL), hyponatremia (Na 127 mmol/L), hypocalcemia (Ca 8.4 mg/dL), and elevated aminotransferases (AST 60 U/L, ALT 66 U/L). BNP was elevated at 128.6 pg/mL, and prothrombin time was 18.3 seconds with an INR of 1.46. Urinalysis showed trace protein with occasional bacteria. Thyroid-stimulating hormone was 7.849 μIU/mL, and troponin and lipase were within normal limits. Laboratory results for two days after discharge are shown in Table 1.

**Figure 5:**
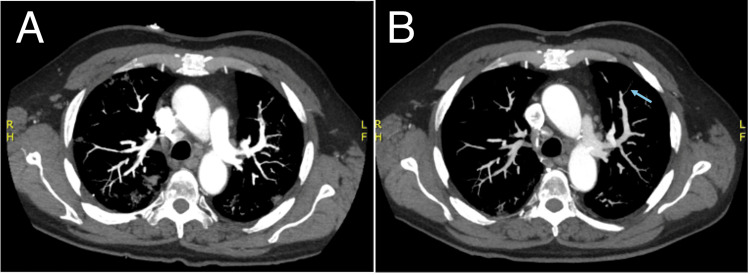
CTA chest comparison. Panel A (left): initial presentation. Panel B (right): readmission two days later demonstrating a new pulmonary embolus in the left upper lobe (blue arrow) compared with Panel A.

CTA of the chest, abdomen, and pelvis with intravenous contrast demonstrated a new lobulated filling defect in a subsegmental branch of the left upper lobe pulmonary artery, consistent with acute pulmonary embolism (Figure 5). Previously identified pulmonary emboli were decreased in size, and interval resolution of the prior left atrial appendage thrombus was noted. Repeat abdominal imaging showed no new splenic infarcts and no interval change in the spleen. Pulmonary findings remained suspicious for pneumonia. Cardiology was consulted, and rate control therapy was adjusted from diltiazem to metoprolol. Given recurrent embolism in the setting of documented nonadherence to prescribed outpatient anticoagulation, therapeutic anticoagulation was resumed. The patient was discharged on apixaban with a loading regimen of 10 mg twice daily for four days followed by 5 mg twice daily, along with metoprolol succinate 100 mg once daily, diltiazem extended release 360 mg once daily, cefdinir 300 mg twice daily for seven days, and a short course of prednisone 40 mg daily for three days.

## Discussion

AF is the most common cardiac arrhythmia worldwide, characterized by rapid atrial electrical activity that increases the risk of thrombotic events and stroke. While cerebral embolic events are more common than systemic emboli, rare complications such as SI are possible. In a 10-year retrospective cohort study, SIs of various etiologies accounted for only 0.016% of inpatient admissions, highlighting their uncommon nature [4]. Many splenic complications remain asymptomatic; when symptoms do arise, they often mimic other abdominal pathologies, creating challenges for diagnosis and timely treatment [5].

In this case, a 60-year-old man with chronic AF, hypertension, and chronic tobacco use developed SI in the setting of anticoagulation instability while maintained on warfarin therapy. Oral anticoagulation with vitamin K antagonists or direct oral anticoagulants significantly reduces thromboembolic risk in AF [11], yet thromboembolic events may still occur in clinical practice when anticoagulant exposure is inconsistent. The clinical course was further complicated by a left atrial appendage thrombus, a recognized embolic source in AF, and by recurrent pulmonary embolism following discharge in the setting of documented nonadherence to prescribed apixaban therapy. Prior evidence suggests that preceding antithrombotic therapy may influence ischemic stroke severity and in-hospital outcomes among patients with AF, reinforcing that thromboembolic risk persists in real-world settings and may not be eliminated by prescribed therapy alone [14]. Suboptimal anticoagulation may result from inadequate therapeutic monitoring, fluctuating INR control, drug interactions, variable pharmacokinetics, and lapses in adherence, all of which contribute to preventable anticoagulant-related complications [11,12]. In addition, surgical management is generally reserved for select splenic complications such as rupture, abscess, or extensive necrosis and is not routinely required in hemodynamically stable patients [15]. Despite bleeding risks, appropriate anticoagulant therapy has been associated with improved long-term outcomes and decreased mortality in patients with SI [16].

AF promotes thrombus formation primarily in the LAA, from which clots may embolize to the cerebral or systemic circulation. This prothrombotic state follows Virchow’s triad: stasis, endothelial injury, and hypercoagulability [10]. Hypokinetic atrial contractions in AF cause blood pooling in the LAA, whose impaired contractility increases thrombus susceptibility [9]. While cerebral infarcts are more common due to the direct path from the aorta to the carotids [17, 18], and thromboembolism in AF predominantly affects the cerebral circulation [19], systemic embolization to the splenic artery can also occur, causing splenic ischemia and necrosis [1,4,5].

Anticoagulation failure is often multifactorial. In this patient, an INR of 1.98 below the recommended 2.0-3.0 therapeutic range was likely a major contributor [13]. Suboptimal INR can result from inadequate dosing, poor adherence, drug interactions, or anticoagulation resistance as demonstrated in cohort studies [11,14]. This patient’s history of recurrent subtherapeutic INR readings while on warfarin for chronic AF warrants evaluation for underlying causes, including financial barriers, limited follow-up, or resistance to anticoagulation.

In Schattner et al.’s 10-year study, 75% of hospitalized SI patients reported diffuse abdominal pain, while fewer than one-third had no pain, illustrating its variable presentation [4]. By contrast, ischemic strokes from AF have a far higher incidence (1-20%) [7] and are diagnosed more readily due to their acute neurological warning signs. SI symptoms include left upper quadrant pain, fever, chills, and shoulder pain, which can mimic other abdominal conditions and delay diagnosis [1]. These findings support including SI in the differential for acute abdominal pain in AF patients.

Contrast-enhanced CT is the gold standard for diagnosing SI, showing characteristic wedge-shaped hypodensities [1,5]. In our patient, early CT confirmed the diagnosis. Given the coexistence of pulmonary embolism, treatment included IV heparin bridged to warfarin to rapidly achieve anticoagulation, especially in the setting of a subtherapeutic INR. Splenectomy was not indicated because the patient remained hemodynamically stable; surgical intervention is generally reserved for rupture or extensive necrosis [15].

The patient’s concurrent pneumonia, with gram-positive bacilli bacteremia, suggests a possible hypercoagulable state triggered by infection [20]. Although it is unclear whether this contributed to his splenic infarct, infections are known to promote thrombosis. Broad-spectrum antibiotics (piperacillin-tazobactam) were started to address the pneumonia and reduce the risk of secondary complications such as splenic abscess.

This case highlights the importance of maintaining a high index of suspicion for systemic emboli in AF patients, even when they are anticoagulated. Vague abdominal symptoms should prompt timely imaging to avoid missed or delayed diagnoses. Personalized anticoagulation regimens and close therapeutic monitoring are essential in preventing recurrent embolic complications. Further research on anticoagulation resistance and individualized preventive strategies may improve outcomes for this high-risk group.

## Conclusions

This case report contributes to the literature on anticoagulation management in AF by elucidating the formidable challenges of achieving and sustaining therapeutic stability in complex clinical settings. Despite chronic warfarin therapy, the patient exhibited persistent difficulty maintaining therapeutic INR levels, and following inpatient anticoagulation overlap, he was transitioned to apixaban at discharge; documented nonadherence to outpatient therapy was subsequently associated with recurrent pulmonary embolism in the setting of recent SI and prior left atrial appendage thrombus. The clinical trajectory suggests that variability and discontinuity in anticoagulant exposure, rather than simultaneous pharmacologic failure, may permit ongoing thrombus formation and systemic embolization. His readmission with pneumonia, likely acquired through household exposure, may have further amplified an already prothrombotic milieu during a period of anticoagulation instability. These events call for disciplined therapeutic monitoring, deliberate coordination during transitions between regimens, and patient-centered counseling that prioritizes adherence and early symptom recognition. More broadly, this case reinforces the imperative for individualized anticoagulation strategies that account for comorbid illness, social context, and continuity of care. Continued investigation into the interplay between acute infection, treatment transitions, and patient-specific factors may inform more precise and personalized management approaches, with the potential to meaningfully reduce morbidity and mortality among high-risk AF populations.
